# Diffraction response of photorefractive polymers over nine orders of magnitude of pulse duration

**DOI:** 10.1038/srep29027

**Published:** 2016-07-01

**Authors:** Pierre-Alexandre Blanche, Brittany Lynn, Dmitriy Churin, Khanh Kieu, Robert A. Norwood, Nasser Peyghambarian

**Affiliations:** 1 College of Optical Sciences, The University of Arizona, Tucson AZ, USA

## Abstract

The development of a single mode fiber-based pulsed laser with variable pulse duration, energy, and repetition rate has enabled the characterization of photorefractive polymer (PRP) in a previously inaccessible regime located between millisecond and microsecond single pulse illumination. With the addition of CW and nanosecond pulse lasers, four wave mixing measurements covering 9 orders of magnitudes in pulse duration are reported. Reciprocity failure of the diffraction efficiency according to the pulse duration for a constant energy density is observed and attributed to multiple excitation, transport and trapping events of the charge carriers. However, for pulses shorter than 30 μs, the efficiency reaches a plateau where an increase in energy density no longer affects the efficiency. This plateau is due to the saturation of the charge generation at high peak power given the limited number of sensitizer sites. The same behavior is observed in two different types of devices composed of the same material but with or without a buffer layer covering one electrode, which confirm the origin of these mechanisms. This new type of measurement is especially important to optimize PRP for applications using short pulse duration.

Photorefractive polymers (PRP) are organic compounds that dynamically change their index of refraction upon illumination due to charge photogeneration, transport, trapping and molecular reorientation in the local space-charge field. This effect is at the core of many applications such as phase conjugate mirrors, image correlation, wave mixing, optical amplification, and the holographic 3D display^1^.

First discovered in 1991 by Ducharme *et al*.^2^, PRP have been studied in detail and highly optimized for more than two decades. The various aspects of their behavior such as the diffraction efficiency, grating phase shift, charge photogeneration and trapping dynamics has enabled the development of a comprehensive theoretical model explaining the different microscopic aspects of the material. Initially developed for photorefractive inorganic crystals by Moharam *et al*.^2^,^3^, as well as Kuktarev *et al*.^4^,^5^, the dynamic model of the space-charge field was subsequently enriched to take into account the specific mechanisms observed in organic and polymer systems by Schildkraut *et al*.^6^,^7^, Ostroverkhova *et al*.^8^, and Oh *et al*.^9^, who included two trap energies (shallow and deep) to better reproduce the photoconduction dispersion. Moerner *et al*.^10^. explained the unusually high value of the index modulation observed in PRP by including an orientational enhancement effect that produces birefringence in addition to the hyperpolarizability of the chromophore molecules.

The reliability of this model has been put to the test numerous times and it has been able to interpret different observed behaviors in PRP such as the dynamics of the diffraction efficiency due to trap energy^11^,^12^ or gain reversal due to charge carrier competition^13^,^14^. The deep understanding of the microscopic phenomena responsible for the photorefractive effect in polymers has led to multiple scale factors of improvement in the different figures of merit of these materials such as the diffraction efficiency^15^, sensitivity^16^,^17^, and gain^18^ that has allowed the pursuit of an increased variety of applications.

During the development of a holographic 3D display, our team has observed a dramatic reduction of the diffraction efficiency by a factor of 50 in PRP when illuminated with a nanosecond pulsed laser^19^ instead of CW^20^. In both experiments, the material was illuminated by the same energy density (20 mJ/cm^2^), but the delivery time and peak power were very different (by a factor 10^9^). This phenomenon known as “reciprocity failure” has already been observed in permanent holographic recording materials such as silver-halide^21^, dichromated gelatin^22^, and photopolymer^23^, and is intrinsically linked to the electronic response of the material to illumination. So far, and to the best of our knowledge, it has never been explained in PRP, and is not taken into account in the prevalent model introduced above.

The desire for increased understanding of reciprocity failure in PRP comes not only from a need for a better knowledge of the underlying electronic mechanisms, but also for optimization of the sensitivity and efficiency of the material according to the recording intensity available with current laser technology. This optimization is needed in many applications so that the recording source power and volume can be reduced to a minimum. Until recently, for 532 nm wavelength operation, the available laser sources were limited to CW or Q-switched operation with a few nanoseconds of pulse duration. The development of a new, compact single mode fiber-based laser has enabled the characterization and optimization of PRP devices across previously inaccessible region of pulse durations from 250 ns to 1 ms. By analyzing the time response and diffractive performance of the current PRP systems in this new pulse duration range, it is possible to operate the laser in a region with the best material response, or conversely to optimize the material for a specific pulse duration where the laser is the most effective.

In this paper we present a new compact laser source with tunable pulse width and a useful energy per pulse for PRP based applications, discuss the dynamic and maximum diffraction efficiency of two PRP systems according to the temporal pulse width of the writing beams spreading 9 orders of magnitude from nanosecond to seconds, and propose a model that explains the observed behavior.

## Experimental Section

### Material

**Figure 1:**
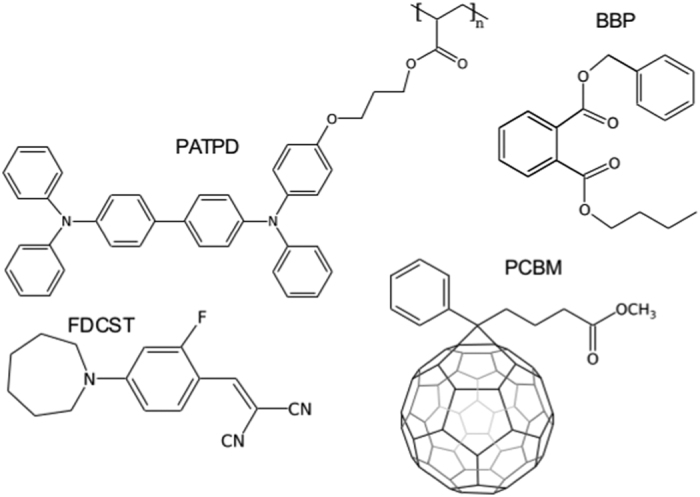
Molecular structures of the different compounds used in the PRP devices.

The PRP material used in this study is based on a modified polyacrylic-tetraphenyldiaminobiphenyl (PATPD) in which carbaldehyde aniline (CAAN) was attached to the PATPD copolymer chain in a ratio of 10:1 (TPD:CAAN). This additional component reduces the tendency of the low T_g_ guest-host system to phase separate, increasing the loading limit of the chromophore, fluorinated dicyanostyrene 4-homo-piperidino benzylidine malononitrile (FDCST)^17^,^19^,^24^. The plasticizer is benzyl butyl phthalate (BBP), and the sensitizer was [6,6]-phenyl-C61-butyric acid methyl ester (PCBM). The different components were combined in a ratio of TPD:CAAN/FDCST:BBP:PCBM (56.23:33.74:9.84:0.2 wt%). The molecular structures of these compounds are presented in Fig. 1 .

For the first device we studied, the solution of the different components was dried in a powder which was heated and pressed between two transparent indium tin oxide (ITO) electrodes on glass to a thickness of 100 μm.

The second device we used was composed of the same material with the same thickness, but a 10 μm thick buffer layer of amorphous polycarbonate (APC) was spin coated on top of one ITO electrode. Such buffer layers are known to dramatically change the properties of the PRP device due to the build-up of charges at the interface, decreasing the number of trapped charges, and reducing the current^12^,^24^,^25^. The buffer layer allows operation of the device at a lower applied voltage due to the displacement of the diffraction efficiency over-modulation peak to a lower voltage, which reduces the chance of dielectric breakdown and enhances the lifetime of the device. This makes the comparison between the two systems relevant for both material and application driven research.

### Laser sources

Three different laser sources were used in this study. The first one is a CW diode-pumped solid-state laser (Coherent Verdi V18). This laser, which emits at 532 nm, was externally chopped with a Pockels cell to achieve a range of exposure times from milliseconds to seconds. The maximum of 18 watts of CW power available from this source limited the available energy density for the recording beams. For a 1 ms pulse width, the energy at the source is only 18 mJ which is further reduced by losses in the experimental setup. We kept the energy density constant at the sample position through the different laser sources at either 10, 20 or 30 mJ/cm^2^ per pulse, consequently the minimum pulse duration achievable with this CW laser source was 1, 2 and 3 ms respectively.

The shortest time scale measurements were performed using a seeded 6 ns 532 nm InnoLas SpitLight Q-switched laser with 50 Hz repetition rate. The energies of this laser system ramp up to 200 mJ per pulse. To ensure pulse coherence and holographic recording with a single pulse, the pulse selection was achieved with an external Pockels cell.

**Figure 2:**
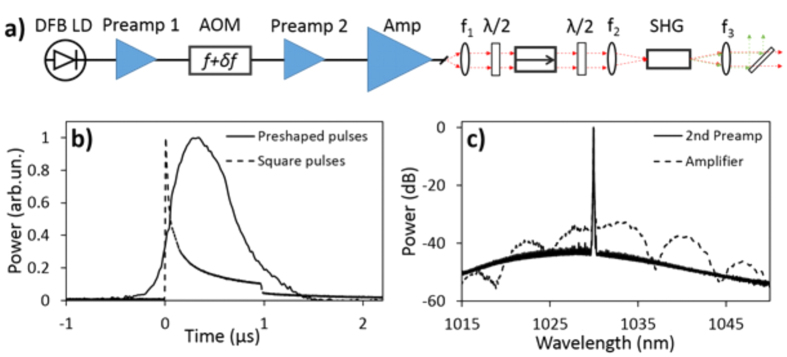
(**a**) Schematic diagram of the MOPA fiber laser system. (**b**) Comparison of the optical pulse shape after amplification for the case of the square (dashed line) and pre-shaped (solid line) electrical pulses driving the AOM. (**c**) Laser spectrum measured after the second fiber preamplifier (solid line) and after the double-clad fiber amplifier (dashed line).

Since there was no commercially available solution that satisfied the power and coherence requirements to access the region between millisecond and nanosecond pulse width, we developed the third laser source. A schematic diagram of the laser system is shown in Fig. 2a, and is based on a master oscillator power amplifier (MOPA)^26^. The seed signal was provided by a distributed feedback laser diode (DFB LD) generating 6 mW of continuous wave (CW), single frequency light at 1030 nm. This signal was amplified to 200 mW in a ytterbium-doped fiber preamplifier (Preamp 1). At this point, the output was modulated into pulses using a fiber-coupled acousto-optic modulator (AOM) with a repetition rate tunable from 3–10 kHz. The form of the electrical signal driving the AOM was designed to counter the distortion from the following amplifier stages and produce optical pulses with a nearly Gaussian pulse shape. Figure 2b shows the resulting pulse shape for a square wave driving voltage (dashed line) and the optimized pulse (solid line). The power of the modulated optical signal after the AOM varies with respect to the pulse duration and repetition rate from 0.1 mW to 50 mW. A second ytterbium-doped fiber preamplifier and a ytterbium-doped double clad fiber amplifier scale the modulated optical signal to 100 mW and 5 W, respectively. At the maximum repetition rate of 10 kHz and minimum pulse duration of 200 ns, the spontaneous emission after the second preamplifier and the main amplifier stages was measured to be 3% and 13%, respectively (spectra shown in Fig. 2c). This has a minimal effect on the final laser performance. After amplification, the optical pulses were collimated using an aspheric lens followed by a high-power free-space isolator. The 1030 nm optical pulses are frequency doubled through second harmonic generation (SHG) using a 10 mm periodically poled magnesium-doped stoichiometric lithium tantalite (MgSLT) nonlinear crystal. The resulting optical pulses at 515 nm were collimated, and the residual pump was stripped with a dichroic mirror. The final maximum average optical power at 515 nm and 3 kHz repetition rate ranges from 500 mW for 200 ns pulses to 1.2 W for 1 ms pulses. The coherence length of the emitted pulses was measured with an interferometer to be 2 cm which prescribed the need for a time delay line to equalize both arms of the four-wave mixing experiment.

### Four-Wave Mixing

We measured the diffraction efficiency dynamic response of single pulse holographic recording using a non-degenerate four-wave mixing (FWM) setup. Details of the setup can be found eleswhere^27^. The probe beam is provided by a 633 nm HeNe which is *p*-polarized to maximize the diffraction efficiency due to the larger index modulation experienced in this configuration. The optimal Bragg readout angle was obtained experimentally by maximizing the diffraction efficiency of the probe beam when a grating was recorded continuously with multiple pulses. The recording beams come from one of the three different laser sources described in the previous section and are focused at the sample location. The energy density was calculated according to an energy measurement in front of the sample, and the waist diameter derived from beam diameter and lens focal lengths. The absorption coefficient of the photorefractive material (both devices) at 532 nm is 140 cm^−1^, which makes this wavelength appropriate for recording the grating pattern. No material damage was observed at the level of power density used during the experiments. At 633 nm the absorption coefficient is only 6 cm^−1^ allowing for good transmission and minimal perturbation of the grating during reading. Incidence angles were set to 30° and 65° in air to obtain a large slant angle inside the material and achieve a large projection of the grating vector onto the external electric field. To maximize the fringe contrast, the recording beams were *s*-polarized, the beam ratio inside the sample was set to 1:1 (2:1 outside the sample considering the different beam cross-sections and Fresnel reflection), and a delay line ensured that both beam paths have the same length.

## Results and Discussion

**Figure 3:**
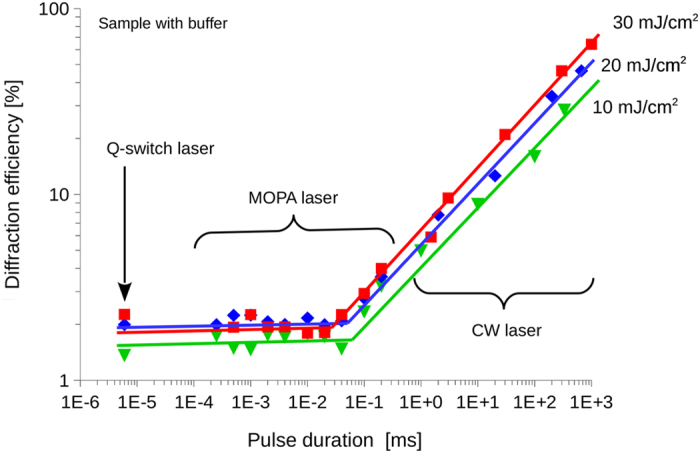
Single pulse diffraction efficiency according to writing pulse duration for various energy densities per pulse. Sample with buffer layer at 42.5 V/μm. Square 30 mJ/cm^2^, diamond: 20 mJ/cm^2^, triangle 10 mJ/cm^2^. Lines are independent interpolations of the plateau and slope regions.

The diffraction efficiency measurements presented in Fig. 3 were made on the sample with a buffer layer at an applied voltage of 42.5 V/μm. Single pulse recording of a diffraction grating was monitored with the probe beam and the maximum value is reported according to the pulse duration. Three different values of the energy density per pulse were used: 10, 20, and 30 mJ/cm^2^.

Given that the energy density per pulse is kept constant over the measurement for the different traces, we expected to observe no change in the diffraction efficiency. On the contrary, reciprocity failure can be observed with a dramatic reduction of the diffraction efficiency with the pulse duration. The dependence of the diffraction efficiency on the pulse duration follows a power law until 30 μs where the reciprocity failure stops and a plateau is reached for values of the pulse duration below 30 μs. This indicates a change of regime in the space-charge field buildup. Since the exact same behavior is observed for each value of the power density it must be intrinsically linked to material properties.

To confirm that the transition between the flat and the increasing efficiency regimes is an inherent material property, we made similar measurements on a second set of samples without buffer layers. In addition to the presence or absence of the buffer layer, the applied voltage was different for the two devices. In the case of the buffered samples, the optimal voltage operation for steady state recording (multiple pulses or CW) was measured to be 42.5 V/μm. At this value, the efficiency reached 92%. Increasing the bias voltage above this point causes the refractive index modulation to grow beyond the optimal Bragg conditions, and the efficiency decreased. Without the buffer layer, the electric field for optimal operation under the otherwise identical recording conditions increased to 72.5 V/μm.

**Figure 4:**
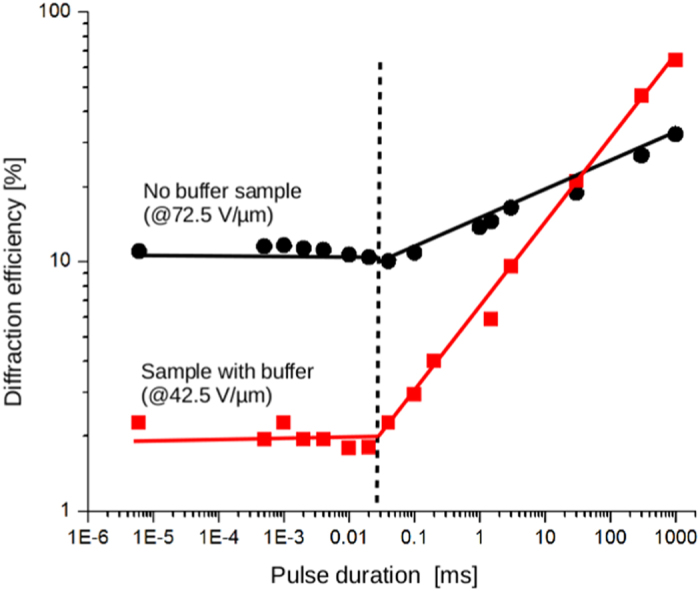
Diffraction efficiency according to the pulse duration for samples with buffer layer (red square) or without buffer layer (black circles). Pulse energy 30 mJ/cm^2^. Lines are guides for the eye.

Comparison between the measurements taken on devices with or without a buffer layer is presented in Fig. 4. Despite the very different voltages and device performance in the steady state regime, the single pulse measurements show that the transition between the plateau and the slope in the diffraction efficiency happens at the same value of pulse duration for both devices: 30 μs.

It can also be seen in Fig. 4 that, while the efficiency without the buffer layer is smaller at large pulse duration (>30 ms) than when the buffer layer is present, the trend is reversed at smaller pulse duration (<30 ms) with an efficiency 5 times larger without the buffer than with the buffer for the plateau region lying between 30 μs and 6 ns. This is particularly important for the optimization of the PRP device for fast refresh rate applications.

**Figure 5:**
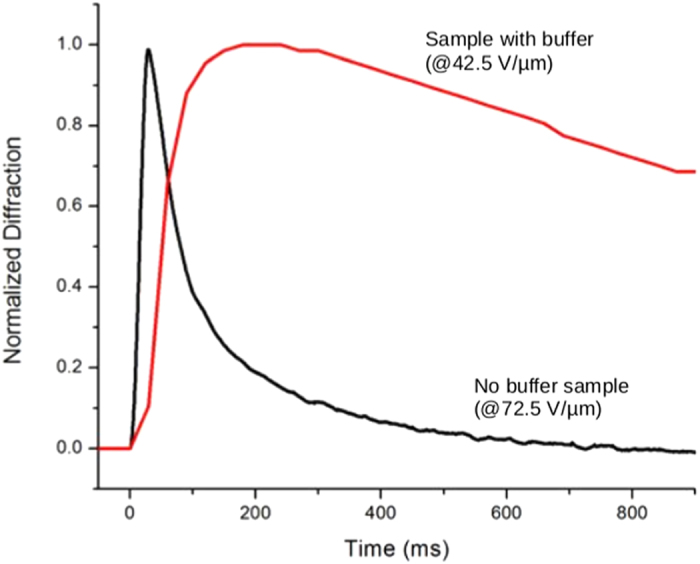
Normalized diffraction efficiency dynamics of devices with buffer layer (red) and without buffer layer (black) in response to a single pulse illumination of 30 mJ/cm^2^ and 10 μs pulse duration. Respective efficiency is 2% with buffer and 10% without buffer (see Fig. 4).

The presence of the buffer and the reduction of the external field also dramatically impact the dynamics of the diffraction as it is presented in Fig. 5. For pulse duration below 30 μs, the speed of the material response without buffer sees a 5 × improvement (faster), with the time of grating formation decreasing from 82 ms to 16 ms. These response times remain constant down to 6 ns pulse duration.

Table 1 summarizes the different parameters used and the measured coefficients from our experiments.

To understand the different behaviors we observed, we need to look back at the three primary time-dependent phenomena contributing to the development of a refractive index grating: photogeneration, charge transport and trapping, and chromophore orientation to the space-charge field.

The photogeneration of charges in response to the incident illumination takes on the order of picoseconds^28^. This is three orders of magnitude faster than the nanosecond pulses used in our experiment, indicating that the process by itself should not be significantly affected by the pulse durations under the present investigation. It is only for pulses with much shorter duration such as the femtosecond regime that new processes such as two-photon absorption becomes significant^29^, a domain not under consideration in the present study. On the other hand, photogeneration can be saturated by a large peak power, effectively using up the fixed number of sensitizer sites available at the onset of illumination. Such saturation phenomena have previously been observed for the photocurrent of photoconducting polymers^30^, as well as for the charge carrier density of photorefractive crystals^31^. If saturation indeed occurs, increasing the power density of the writing beams does not further increase the efficiency. That is exactly the behavior observed in Fig. 3 for the plateau region up to the millisecond regime: when the pulse energy density is increased from 10 to 30 mJ/cm^2^, there is no obvious increase in diffraction efficiency. It is only for pulse durations larger than 10 ms that an increase of energy density clearly results in an increase of the diffraction efficiency as can be seen from the data interpolation in this region.

For a saturated process, a shift in the onset of the transition between the plateau and the increase of the efficiency according to the pulse duration is expected when the pulse energy density is increased. This shift cannot be directly observed in the data since it is masked by the standard deviation of the measurement. However, the linear interpolation of the long pulse data points (>10 ms) and the short data point (<30 μs) presented in Fig. 3 indicates that a shift is indeed present.

Since the composition of both devices we used is identical, especially the sensitizer concentration, the saturation behavior should be the same regardless of the presence or absence of the buffer layer, as is born out in Fig. 4. However, the photogeneration efficiency is strongly field dependent, as expressed in the Onsager formalism^32^,^33^, and the fact that the samples without buffer operates at larger external fields explains the large efficiency observed for this type of device at shorter pulse durations.

The second mechanism in the photorefractive process is the transport and trapping of the now excited charge carriers. The characteristic times for these events are on the order of 0.1–5 ms, well within the range of pulse duration that our FWM experiment is probing^34^. Transport speed (mobility) and trapping rates are also highly field dependent and are expected to play a role in the buffer/no-buffer device comparison.

**Figure 6:**
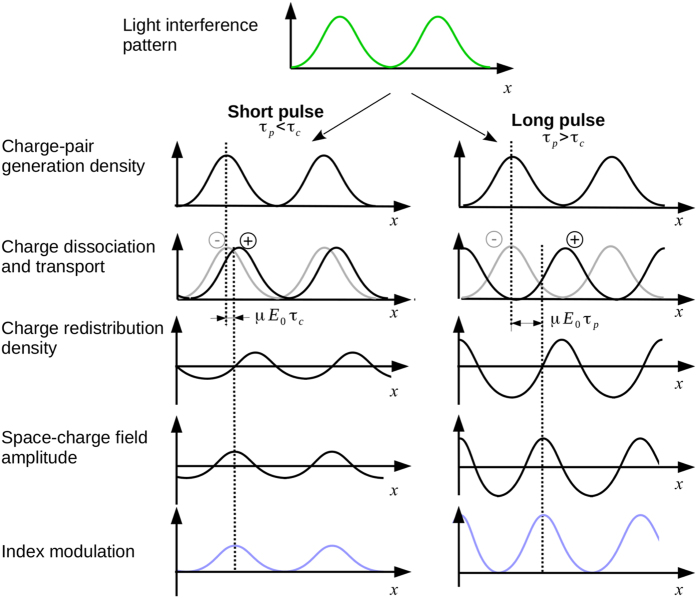
Photorefractive mechanism for short (left) or long (right) pulse durations. See text for explanation.

The following reasoning can be applied: for pulse durations shorter than the lifetime of the charge carriers, there is a single event of excitation, transport, and trapping. Once trapped, the charges are no longer mobile since there is no additional light to re-excite them. Under these conditions, the charge separation (*φ*) is at a minimum and is given by the lifetime of the carriers (*τ*_*c*_) multiplied by their mobility (*μ*) and external field (*E*_0_): *φ* = *μE*_0_*τ*_*c*_. This behavior is depicted in the left panel of Fig. 6.

As long as the pulse duration is shorter than the lifetime of the charge carrier, there is no modification of the charge separation distance. This leads to a constant, space-charge field, index modulation, and diffraction efficiency. This regime of operation can explain the plateau present in our measurement bellow 30 μs pulse duration. However, it cannot explain by itself the constant diffraction efficiency observed according to the pulse intensity in the plateau region. For this reason, the saturation of charge generation we discussed earlier is also included.

As the pulse duration *τ*_*p*_ increases over the lifetime of the charge carriers, the illumination can support multiple excitation, transport, and trapping events for each carrier^35^. The distance traveled by the carriers becomes larger and proportional to the pulse duration:


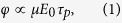


with larger separation of the carriers, recombination is less likely and the space-charge field can increase. This behavior is depicted in the right panel of Fig. 6.

It can be argued that the number of independent charges generated with long pulses is proportionally smaller due to the multiple excitations of the same carriers that have been trapped during the pulse duration. However, we are operating in domain of small absorption where the number of photons is an order of magnitude larger than the sensitizer density *N*_*A*_. Therefore, the spatial charge density for the non mobile species *ρ*_±_ and the charge carrier 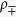 can be expressed as:


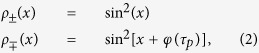


where *x* is the period of the illumination pattern.

The amplitude of the space-charge field is proportional to the difference between the two distributions:


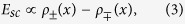


which is linear according to the pulse duration in the region of interest and can explain the increase of the diffraction efficiency observed above the 30 μs pulse duration.

This linear increase of the space-charge field according to the pulse duration should translate into a quadratic increase of the diffraction efficiency in the small index modulation regime:


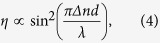


with *d* the thickness of the sample, and *Δn* the index modulation which, in the case of birefringence dominated PRP, is related to the space-charge field by^10^:


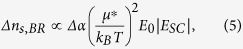


with *Δα* the difference between the polarizabilities along and perpendicular to the chromophore molecular axis, μ^*^ the permanent dipole moment of the chromophore, *k*_*B*_ the Boltzmann’s constant, and *T* the temperature. Constant geometric factors have been removed from both equations 4 and 5 for conciseness.

While the measurements show a clear increase with the pulse duration, the coefficients are not quadratic but sub-linear:


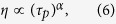


with *α* = 0.34 for the device with buffer layer, and *α* = 0.11 for the device without buffer. This could be due to a more complicated interaction between the charges and the traps than foreseen by the theory developed here which assumes steady state performance. It is well-known that photorefractive polymers experience a large dispersion of the charges during photoconduction and that traps of different energy levels are present^8^,^36^. Both factors influence the space-charge field, and a more elaborate model is needed to describe these behaviors more accurately. However, the set of differential equations taking into account the time dependency are not analytically solvable (no closed-form solution).

The final step for the formation of the diffraction grating in PRP is the chromophore orientation to the space-charge field. This mechanism takes on the order of 0.1 to 1 ms^37^. Since the temporal scale is within the pulse duration we are using for the writing beams, the orientation process is not expected to be dependent on the illumination process since the molecular reorientation occurs in response to the space-charge field and not the light field as in the case of pure non-linear optical processes.

The fact that the reciprocity failure could be due to a limited number of excitation sites indicates that the sensitizer loading which was optimized for the CW regime may be too low for optimal pulsed operation. Future experiments will include similar measurements with different type of PRPs such as PVK matrix based system since this material has very different dynamics^38^.

## Conclusions

Thanks to the development of a new laser with adjustable pulse duration, the diffraction efficiency in photorefractive polymer systems has been measured for the first time over 9 orders of magnitude of writing beam pulse duration at constant energy densities. Reciprocity failure has been observed in which reducing the pulse duration reduces the diffraction efficiency according to a power law. Below 30 μs, the diffraction efficiency reaches a plateau and does not decrease further. In this plateau region, increasing the writing beam energy density does not increase the diffraction efficiency indicating a saturation phenomenon.

Measurements of two photorefractive types of device composed of the same polymer but with or without a buffer layer deposited over an electrode, showed the same behavior with the plateau starting at the exact same pulse duration (30 μs). This is a strong evidence that the mechanism is due an inherent material property that we linked to the lifetime of the charge carriers *τ*_*c*_.

Two hypotheses based on the current knowledge of the PR mechanism are advanced to explain the observed behaviors: saturation of the charge generation at high peak power given the limited number of sensitizer sites, and single versus multiple excitation, transport and trapping events of the charge carriers according to the pulse duration.

This new type of measurement of the diffraction efficiency according to the pulse duration is quite valuable, not only for a better understanding of the photorefractive mechanism in polymers by itself, but also for the optimization of PRP systems for pulse driven applications such as video rate holographic 3D display, image processing, correlation, or phase conjugation at high frame rates.

In closing, the ability to tune the pulse duration over a large range of operation opens a brand new domain of exploration for PRP material with a high potential for discovery since the classical theory of photorefractivity in polymers does not incorporate the type of behaviors we have encountered.

## Additional Information

**How to cite this article**: Blanche, P.-A. *et al*. Diffraction response of photorefractive polymers over nine orders of magnitude of pulse duration. *Sci. Rep.* **6**, 29027; doi: 10.1038/srep29027 (2016).

## Figures and Tables

**Table 1:** Parameters and coefficients for the different photorefractive devices used in the experiment.

Sample	Optimal field [V/μm]	Efficiency behavior transition	Efficiency < 30 ms	Efficiency at 1000 ms	Time constant at 10 μs
With buffer	42.5	30 ms	2%	65%	65 ms^−1^
Without buffer	72.5	30 ms	10%	30%	16 ms^−1^

## References

[b1] Blanche P.-A. Photorefractive Organic Materials and Applications. (Springer, to be published June 2016).

[b2] Ducharme S. P., Scott J., Twieg R. & Moerner W. Observation of the photorefractive effect in a polymer. Phys. Rev. Lett. 66, 1846 (1991). 10043325 10.1103/PhysRevLett.66.1846

[b3] Moharam M. G. & Young L. Hologram writing by the photorefractive effect. J. Appl. Phys. 48, 3230–3236 (1977).

[b4] Kukhtarev N., Markov V. & Odulov S. Transient energy transfer during hologram formation in LiNbO3 in external electric field. Opt. Commun. 23, 338–343 (1977).

[b5] Kukhtarev N. V., Markov V. B., Odulov S. G., Soskin M. S. & Vinetskii V. L. Holographic storage in electrooptic crystals. I. Steady state. Ferroelectrics 22, 949–960 (1979).

[b6] Schildkraut J. S. & Buettner A. V. Theory and simulation of the formation and erasure of space-charge gratings in photoconductive polymers. J. Appl. Phys. 72, 1888–1893 (1992).

[b7] Schildkraut J. S. & Cui Y. Zero-order and first-order theory of the formation of space-charge gratings in photoconductive polymers. J. Appl. Phys. 72, 5055–5060 (1992).

[b8] Ostroverkhova O. & Singer K. D. Space-charge dynamics in photorefractive polymers. J. Appl. Phys. 92, 1727–1743 (2002).

[b9] Oh J. W., Lee C. & Kim N. Influence of chromophore content on the steady-state space charge formation of poly[methyl-3-(9-carbazolyl) propylsiloxane]-based polymeric photorefractive composites. J. Appl. Phys. 104, 0–6 (2008).

[b10] Moerner W. E., Silence S. M., Hache F. & Bjorklund G. C. Orientationally enhanced photorefractive effect in polymers. J. Opt. Soc. Am. B 11, 320 (1994).

[b11] Christenson C. W. . Grating dynamics in a photorefractive polymer with Alq(3) electron traps. Opt. Express 18, 9358–65 (2010). 20588782 10.1364/OE.18.009358

[b12] Liebig C. M. . Achieving enhanced gain in photorefractive polymers by eliminating electron contributions using large bias fields. Opt. Express 21, 30392–400 (2013). 24514617 10.1364/OE.21.030392

[b13] Wang L., Ng M.-K. & Yu L. Photorefraction and complementary grating competition in bipolar transport molecular material. Phys. Rev. B 62, 4973–4984 (2000).

[b14] Banerjee P. P. . Time dynamics of self-pumped reflection gratings in a photorefractive polymer. J. Appl. Phys. 111, 013108 (2012).

[b15] Meerholz K., Volodin B., Sandalphon, Kippelen B. & Peyghambarian N. A photorefractive polymer with high optical gain and diffraction efficiency near 100%. Nature 371, 497 (1994).

[b16] Kinashi K., Wang Y., Sakai W. & Tsutsumi N. Optimization of photorefractivity based on poly(N-vinylcarbazole) composites: An approach from the perspectives of chemistry and physics. Macromol. Chem. Phys. 214, 1789–1797 (2013).

[b17] Eralp M. . Submillisecond response of a photorefractive polymer under single nanosecond pulse exposure. Appl. Phys. Lett. 89, 114105 (2006).

[b18] Suh D. J., Park O. O., Ahn T. & Shim H.-K. Large Two-Beam Coupling in the p-PMEH-PPV/DPP/DO3/C60. Jpn. J. Appl. Phys. 41, L428–L430 (2002).

[b19] Blanche P. . Holographic three-dimensional telepresence using large-area photorefractive polymer. Nature 468, 80–3 (2010). 21048763 10.1038/nature09521

[b20] Tay S. . An updatable holographic three-dimensional display. Nature 451, 694–8 (2008). 18256667 10.1038/nature06596

[b21] Bjelkhagen H. I. Silver-Halide Recording Materials: for Holography and Their Processing. (Springer, 2013).

[b22] Chang B. J. & Leonard C. D. Dichromated gelatin for the fabrication of holographic optical elements. Appl. Opt. 18, 2407–17 (1979). 20212676 10.1364/AO.18.002407

[b23] Zhao G. & Mouroulis P. Diffusion Model of Hologram Formation in Dry Photopolymer Materials. J. Mod. Opt. 41, 1929–1939 (1994).

[b24] Christenson C. W. . Materials for an updatable holographic 3D display. IEEE/OSA J. Disp. Technol. 6, 510–516 (2010).

[b25] Kim W.-S., Lee J.-W. & Park J.-K. Enhancement of the recording stability of a photorefractive polymer composite by the introduction of a trapping layer. Appl. Phys. Lett. 83, 3045 (2003).

[b26] Fang Q. . High power and high energy monolithic single frequency 2 μm nanosecond pulsed fiber laser by using large core Tm-doped germanate fibers: experiment and modeling. Opt. Express 20, 16410 (2012).

[b27] Lynn B., Blanche P.-A. & Peyghambarian N. Photorefractive polymers for holography. J. Polym. Sci. Part B Polym. Phys. 52, 193–231 (2014).

[b28] Haugeneder A. . Exciton diffusion and dissociation in conjugated polymer/fullerene blends and heterostructures. Phys. Rev. B 59, 15346–15351 (1999).

[b29] Blanche P.-A. . Photorefractive polymers sensitized by two-photon absorption. Opt. Lett. 27, 19 (2002). 18007702 10.1364/ol.27.000019

[b30] Lardon M., Lell-döller E. & Weigl J. W. Charge Transfer Sensitization of Some Organic Photoconductors Based on Carbazole. Mol. Cryst. 2, 241–266 (1967).

[b31] Hermann J. P., Herriau J. P. & Huignard J. P. Nanosecond four-wave mixing and holography in BSO crystals. Appl. Opt. 20, 2173–2174 (1981). 20332907 10.1364/AO.20.002173

[b32] Onsager L. Initial recombination of ions. Phys. Rev. 54, 554–557 (1938).

[b33] Mozumder A. Effect of an external electric field on the yield of free ions: General results from the Onsager theory. J. Chem. Phys. 60, 9–13 (1974).

[b34] Maldonado J. L. . Effect of doping with C60 on photocurrent and hole mobility in polymer composites measured by using the time-of-flight technique. Opt. Mater. (Amst). 29, 821–826 (2007).

[b35] Leopold A., Grasruck M., Hofmann U., Kol’chenko M. A. & Zilker S. J. Length scales of charge transport in organic photorefractive materials. Appl. Phys. Lett. 76, 1644 (2000).

[b36] Oh J. W., Lee C. & Kim N. The effect of trap density on the space charge formation in polymeric photorefractive composites. J. Chem. Phys. 130 (2009). 10.1063/1.3103888 19355782

[b37] Herlocker J. A. . Direct observation of orientation limit in a fast photorefractive polymer composite. Appl. Phys. Lett. 74, 2253 (1999).

[b38] Thomas J. . Bistriarylamine Polymer-Based Composites for Photorefractive Applications. Adv. Mater. 16, 2032–2036 (2004).

